# Reaction time-related compression underlies overestimation of time-constrained reachability

**DOI:** 10.1016/j.isci.2026.118115

**Published:** 2026-10-31

**Authors:** Keisuke Tani, Soma Okuuchi, Satoshi Hirose, Keisuke Kushiro

**Affiliations:** 1 Graduate School of Human and Environment Studies, Kyoto University, Kyoto, Japan; 2 Faculty of Psychology, Otemon Gakuin University, Ibaraki, Osaka, Japan; 3 Center for Information and Neural Networks (CiNet), Advanced ICT Research Institute, National Institute of Information and Communications Technology, Osaka, Japan

**Keywords:** action capability, reachability, temporal judgment, motor imagery, reaction time

## Abstract

Humans routinely estimate whether an action can be completed within a limited time window, yet the mechanisms underlying such implicit evaluations are not fully understood. Across three experiments (*N* = 52 in total), we used a psychophysical paradigm to quantify reachability judgments in an arm-reaching task. Participants consistently judged that they could complete the reach even when the available duration was substantially shorter than their actual execution time, indicating an overestimation of time-constrained reachability. This bias contrasts with explicit motor imagery, where imagined durations typically exceed actual execution times. Crucially, by dissociating reaction time (RT) and movement time (MT) components, we show that this overestimation likely arises from selective temporal compression of RT-related component, while MT-related component is largely preserved. Misperception of external duration did not account for the effect. These findings reveal a previously unrecognized cognitive bias and provide new insights into how humans prospectively evaluate their own motor capabilities.

## Highlights

• Time-constrained reachability is overestimated in temporal possibility judgments
• The overestimation mainly arises from compression of the RT-related component
• Target duration perception contributes little to the overestimation

## Graphical abstract

**Figure:**
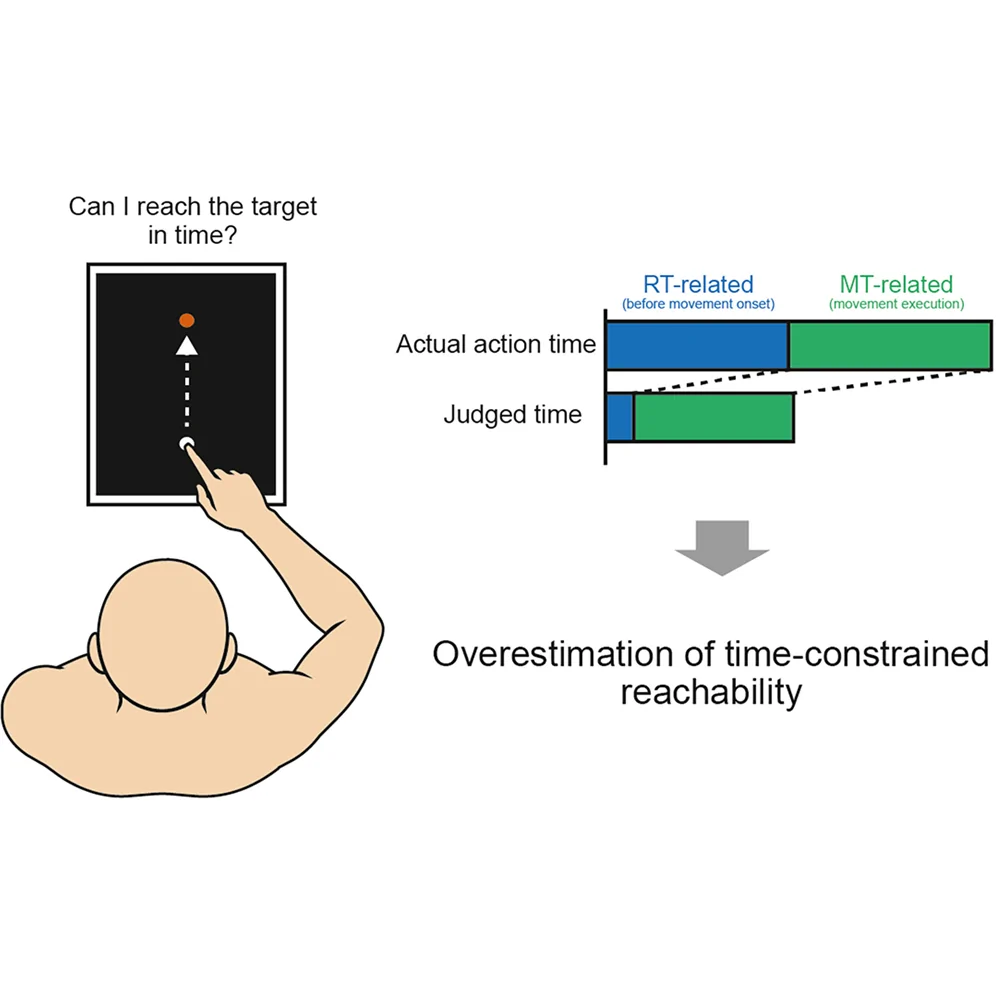

## Teaser

Health sciences; Medicine; Medical specialty; Internal medicine; Neurology; Natural sciences; Biological sciences; Neuroscience; Systems neuroscience; Cognitive neuroscience

## Introduction

In daily life, the ability to judge whether an intended action can be accomplished within a given time frame, referred to here as *temporal possibility judgment of action*, is fundamental to decision-making and subsequent behavior. For example, when crossing a street, if one judges that they cannot reach the other side before the light changes, they will likely wait for the next signal; conversely, if they judge that they can cross in time, they will start walking. Such judgments should require the actor to temporally compare their own action capacity (the time required to reach the other side) with the environmental constraints (the duration of the traffic signal). Although numerous behavioral and neuroscientific findings regarding action capability estimates have accumulated in the context of spatial aspects, such as reachability^1^ and graspability,^2^ to the best of our knowledge, no studies have directly addressed the temporal aspects of such judgments.

Instead, previous studies have mainly investigated the temporal characteristics of action using the mental chronometry paradigm.^3^ This approach explicitly quantifies the duration of mentally simulated movements and compares this imagined duration with that of physically executed movements. The degree of temporal correspondence between imagined and executed movements is commonly used to assess how accurately actual movements are temporally simulated during motor imagery. Findings across various types of actions, such as reaching for an object or walking a certain distance, show that imagined duration is comparable to the duration of actual movements^4^^,^^5^^,^^6^^,^^7^^,^^8^ or longer than them.^9^^,^^10^^,^^11^^,^^12^^,^^13^^,^^14^ Given these findings, one would predict that the capability of time-constrained action would be accurately estimated or, more likely, underestimated (i.e., participants would judge the action as taking longer to complete than the duration actually required). However, this assumption requires further scrutiny for two reasons (Figure 1). First, temporal possibility judgment of action seems to rely on an *implicit* motor simulation representation of action,^15^^,^^16^^,^^17^ in that the underlying estimate of how long an action would take is formed without conscious awareness. Thus, this representation may differ fundamentally from the duration of *explicitly* imagined movement assessed by the mental chronometry paradigm.^18^^,^^19^ Second, temporal possibility judgment is not solely based on this internal representation; it also depends on the representation of the duration of external event timing and the process of temporal comparison between one’s own actions and external events. Therefore, it may not be straightforward to generalize findings from mental chronometry to temporal possibility judgments of action.

**Figure 1:**
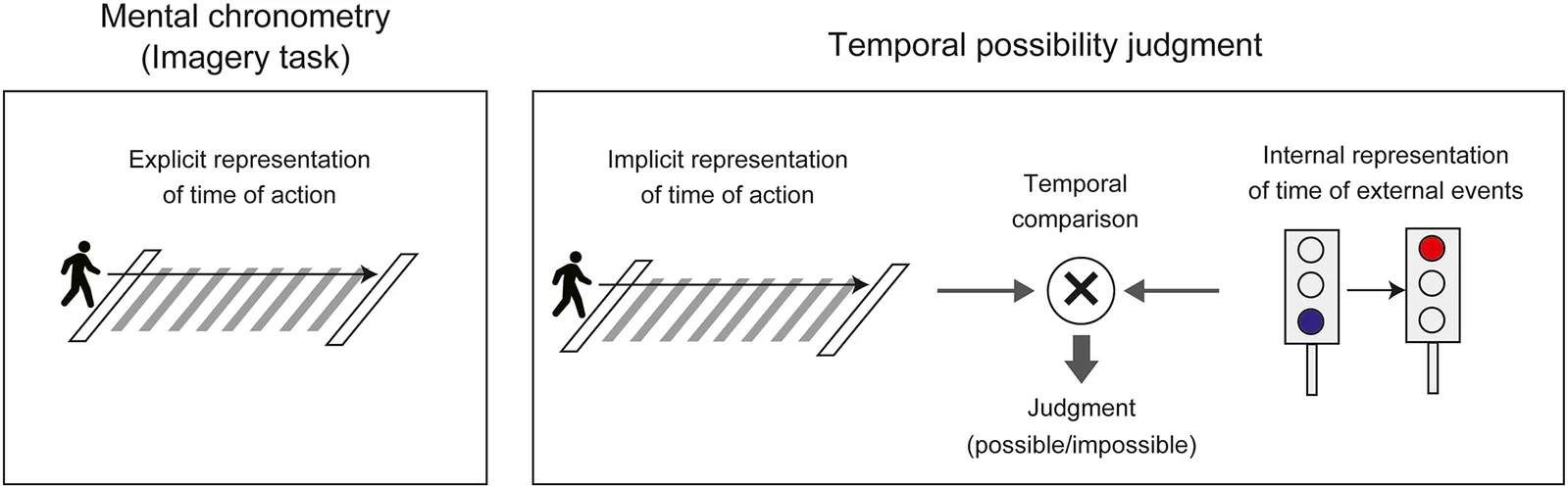
Conceptual illustration of temporal possibility judgment of action To decide whether to cross the street, an individual must implicitly estimate the duration of their own action (time to reach the other side) and compare it with the external temporal constraint (duration of the traffic signal). This process differs from the mental chronometry paradigm, which explicitly requires participants to imagine and reproduce action durations. Temporal possibility judgment also relies on an implicit representation of action duration and its comparison with the duration of an external event.

Here, we report the first systematic investigation of temporal possibility judgment of action using an arm-reaching paradigm. Across three experiments, we introduced a psychophysical paradigm in which participants judged whether a reaching action could be completed within an externally imposed time limit. The central question was whether participants accurately estimate the time required to complete an action when judging action possibility under temporal constraints. The novelty of this paradigm lies in its ability to quantify the temporal boundary of perceived action possibility on the same scale as actual and imagined movement durations. Unlike conventional mental chronometry tasks, which require participants to explicitly report an imagined movement duration, the present paradigm estimates the duration implicitly attributed to an action from binary possibility judgments. This paradigm enables direct comparison among judged, actual, and imagined action durations and allows potential sources of judgment bias to be examined.

The participants judged that they could complete the reach even when the available duration was substantially shorter than their actual execution time (i.e., overestimation of time-constrained reachability), by contrast with the prolonged duration of imagined movements (experiment 1). Importantly, this overestimation of reachability could be primarily attributed to compression of the time required for reaction to the target onset, rather than the time required for movement execution itself (experiment 2). We further ruled out the possibility that the perception of the duration of external events (here, target presentation) contributes to this overestimation (experiment 3). Behavioral analyses supported the validity of the paradigm by showing that judgments reflected decision difficulty and action-related temporal properties. The present findings reveal a previously unrecognized cognitive bias that may underlie action selection and decision-making under temporal constraints.

## Results

### Overestimation of time-constrained reachability

In experiment 1 (*N* = 14), we evaluated the performance of temporal possibility judgments in the arm-reaching task and compared this with the durations of actual and imagined movement. Participants successively performed three tasks (Figure 2A): action, imagery, and judgment tasks, performing each in a separate block before proceeding to the next. In the action task, participants reached toward a visual target presented on the touch display with the right hand as quickly and accurately as possible. In the imagery task, participants imagined performing the reaching movement to the specified target location and indicated the start and end timing of the imagined movement using a keypad. In the judgment task, participants judged whether they could reach the target within a randomly varied presentation duration (0.1–1.0 s) and indicated whether their response was possible or impossible. Across all tasks, targets appeared at locations near or far from the home position following the disappearance of a warning signal (WS; presented at the target location) after a random interval.

**Figure 2:**
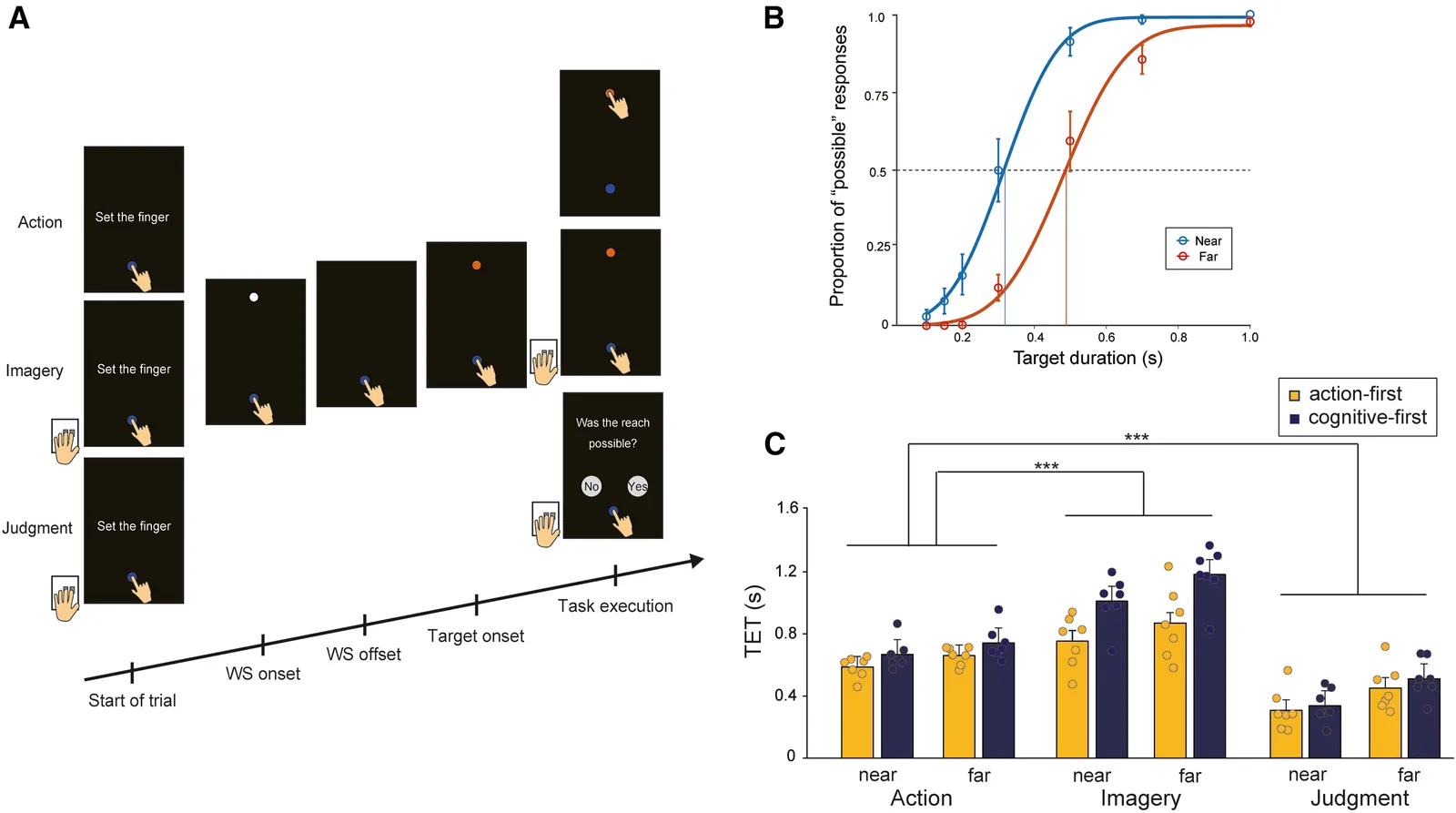
Experimental procedure and behavioral results of experiment 1 (A) Experimental procedure of the temporal possibility judgment task. Participants first placed a finger at the home position and waited for a target to appear. Depending on the block, they (i) actually executed a reach toward the target (action task), (ii) imagined performing the reach without moving (imagery task), or (iii) judged whether the reach would have been possible within the given target duration (judgment task). (B) Group-level psychometric functions for the near and far targets in the judgment task. Circles and error bars represent the mean ± standard error proportion of “possible” responses across participants at each target duration. Curves represent psychometric functions fitted to the pooled responses across participants for visualization purposes. The horizontal dashed line indicates a response proportion of 0.5, and the vertical lines indicate the corresponding PSEs of the pooled fits. Psychometric functions used to estimate PSEs for statistical analyses were fitted separately for each participant and target distance. (C) Group results of experiment 1. Each plot and bar shows the mean TETs for each participant and across participants, respectively. Error bars represent standard deviation. WS, warning signal. ∗∗∗: *p* < 0.001 (paired *t* tests with Bonferroni correction).

For the action and imagery tasks, the reaction time (RT) and movement time (MT) were measured and summed for calculation of the total execution time (TET). For the judgment task, a psychometric function was fitted to the proportion of possible/impossible responses at each target duration, and the point of subjective equality (PSE; the stimulus duration corresponding to a 50% possibility of a response) was calculated as judgment TET. Figure 2B illustrates this procedure using group-level data for visualization purposes. Note that individual PSEs used for statistical analyses were estimated from psychometric functions fitted separately to each participant’s data (see Table S1 for the full set of fitting metrics). The psychometric fits showed high goodness of fit (R^2^: near, 0.99 ± 0.02; far, 0.99 ± 0.01), with mean Weber ratios (WRs) of 0.28 ± 0.14 and 0.24 ± 0.12 for the near and far targets, respectively. A shorter judgment TET indicates that the reaching movement is estimated to take less time. The task order was counterbalanced across participants as a secondary manipulation to assess potential effects of prior motor experience on subsequent cognitive task performance: action → imagery → judgment (action-first group) or imagery → judgment → action (cognition-first group).

Figure 2C shows the TETs for the near and far target in each task. Analyses of variance (ANOVAs) showed significant interactions between task and distance (*F*_1.19, 14.33_ = 8.79, *p* = 0.007, partial eta squared [$\eta_{p}^{2}$] = 0.42) or group (*F*_2, 24_ = 4.32, *p* = 0.03, $\eta_{p}^{2}$ = 0.26; see also Table S2). Post hoc simple main effect analyses revealed that TET was longer for the far target than for the near target in each task (all *p* < 0.05), indicating that the temporal estimates obtained in the imagery and judgment task were sensitive to target distance. Importantly, although imagery TET (near, 0.88 ± 0.21 s; far, 1.03 ± 0.25 s) was significantly longer than action TET (near, 0.63 ± 0.09 s; far, 0.70 ± 0.09 s; all *p* < 0.001, *t*_13_ > 3.20, Cohen’s *d* = 1.21–2.52), judgment TET (near, 0.32 ± 0.12 s; far, 0.48 ± 0.13 s) was significantly shorter than action TET (all *p* < 0.001, *t*_13_ > 4.52, Cohen’s *d* = 1.52–2.11) across both target distances and both groups. These results indicate an overestimation of time-constrained reachability, in that participants judged the reach to be possible within durations substantially shorter than those actually required for movement execution.

A significant task-order effect was observed only in the imagery task. Imagery TETs were longer in the cognitive-first group (near, 1.01 ± 0.16 s; far, 1.18 ± 0.18 s) than in the action-first group (near, 0.75 ± 0.16 s; far, 0.87 ± 0.23 s; *p* = 0.01, *t*_13_ = 3.01, Cohen’s *d* = 1.61). This result is consistent with the previous finding that the duration of imagined action is shortened after the execution of corresponding action.^11^^,^^13^^,^^14^^,^^20^ No significant task-order effect was observed in the judgment task.

To examine whether performing the explicit imagery task before the judgment task contributed to the observed judgment bias (i.e., overestimation of reachability), we also conducted a control experiment with naive participants (*N* = 7) in which the judgment task was performed without a preceding imagery task. Judgment TET remained significantly shorter than action TET (Figure S1), indicating that the overestimation of reachability was not solely attributable to prior engagement in explicit motor imagery.

### Substantial compression of RT-related component for temporal possibility judgment

The results of experiment 1 suggest that the internally represented duration required for movement execution, including reaction to the target onset, is “compressed” during temporal possibility judgment. However, because RT and MT were indistinguishable from judgment TET, it remains unclear to what extent they were compressed. To address this, in experiment 2, temporal possibility judgment was tested under two conditions, one requiring a reaction to the target onset (reaction [R] condition) and one not requiring a reaction (self-initiated [SI] condition), thereby allowing us to infer the relative compression of RT and MT by comparing the TETs between the two conditions.

Participants (*N* = 26) who did not take part in experiment 1 performed the judgment task after the action and imagery tasks. The procedure was identical to experiment 1, but tasks were conducted under both the R and SI conditions. In the R condition, the target appeared at a random time, as in experiment 1, whereas in the SI condition, it appeared when participants released the keypad, eliminating the need for reaction to the target onset. Thus, judgments in the SI condition were expected to reflect only the internally represented movement duration. Task order was fixed (action → imagery → judgment), whereas the R and SI conditions were counterbalanced across participants: R → SI (R-first group) or SI → R (SI-first group).

Table 1 shows the TETs for each task in the R and SI conditions (see also Table S3 for the full set of fitting metrics for the judgment TETs). The psychometric fits showed high goodness of fit (R^2^: R, 0.98 ± 0.04; SI, 0.97 ± 0.04), with mean WRs of 0.30 ± 0.18 and 0.30 ± 0.15 for the R and SI conditions, respectively. ANOVA of the TETs showed significant main effects of task (*F*_1.24, 28.54_ = 24.09, *p* < 0.001, $\eta_{p}^{2}$ = 0.51) and condition (*F*_1, 23_ = 245.18, *p* < 0.001, ${\;\eta }_{p}^{2}$ = 0.91), and a significant interaction between them (*F*_1.23, 28.40_ = 47.31, *p* < 0.001 $,\eta_{p}^{2}$ = 0.67). There was no main effect of group and no interaction between group and task and/or condition (see Table S4 for details). Post hoc simple main effect tests revealed that the TETs in the R condition were significantly longer than those in the SI condition for all tasks (all at *p* < 0.01). Imagery TETs (R condition, 0.96 ± 0.36 s; SI condition, 0.60 ± 0.39 s) were not significantly longer than action TETs (R condition, 0.81 ± 0.11 s, *t*_25_ = 2.08, *p* = 0.15, Cohen’s *d* = 0.41; SI condition, 0.42 ± 0.08 s, *t*_25_ = 2.32, *p* = 0.09, Cohen’s *d* = 0.45). By contrast, judgment TETs (R condition, 0.44 ± 0.22 s; SI condition, 0.36 ± 0.15 s) were significantly shorter than action TETs (R condition, *t*_25_ = 12.49, *p* < 0.001, Cohen’s *d* = 2.45; SI condition, *t*_25_ = 2.90, *p* = 0.02, Cohen’s *d* = 0.57), which replicates the temporal overestimation of reachability in experiment 1.

**Table 1:** Temporal performance of each task under each task condition

	R: RT	R: MT	R: TET (RT + MT)	SI: TET (MT)
Action	0.39 (0.06)	0.42 (0.07)	0.81 (0.11)	0.42 (0.08)
Imagery	0.41 (0.07)	0.55 (0.33)	0.96 (0.36)	0.60 (0.39)
Judgment	N/A	N/A	0.44 (0.22)	0.36 (0.15)

RT, MT, and TET are actual measured values. TET represents the sum of RT and MT where applicable. N/A, not assessed; MT, movement time; RT, reaction time; R, reaction condition; SI, self-initiated condition; TET, total execution time. Values in parentheses indicate standard deviations.

According to the TET data obtained under the two conditions, we operationally estimated RT-related components in each task by subtracting the TET in the SI condition from that in the R condition, whereas we defined MT-related components as the TET in the SI condition (termed *TET-based* RT estimate and MT estimate, respectively; Figure 3A). Both for the action and imagery tasks, TET-based RT estimates (action, 0.39 ± 0.07 s; imagery, 0.36 ± 0.18 s) were relatively similar to the actual RTs measured in the R condition (action, 0.38 ± 0.06 s; imagery, 0.41 ± 0.07 s) and were significantly correlated with the actual RTs across participants (action, *r* = 0.76, *p* < 0.001; imagery, *r* = 0.48, *p* = 0.01). These results confirm that the SI condition successfully captures the motor component of the R condition, implying that participants employed consistent motor planning and execution strategies in both tasks.

**Figure 3:**
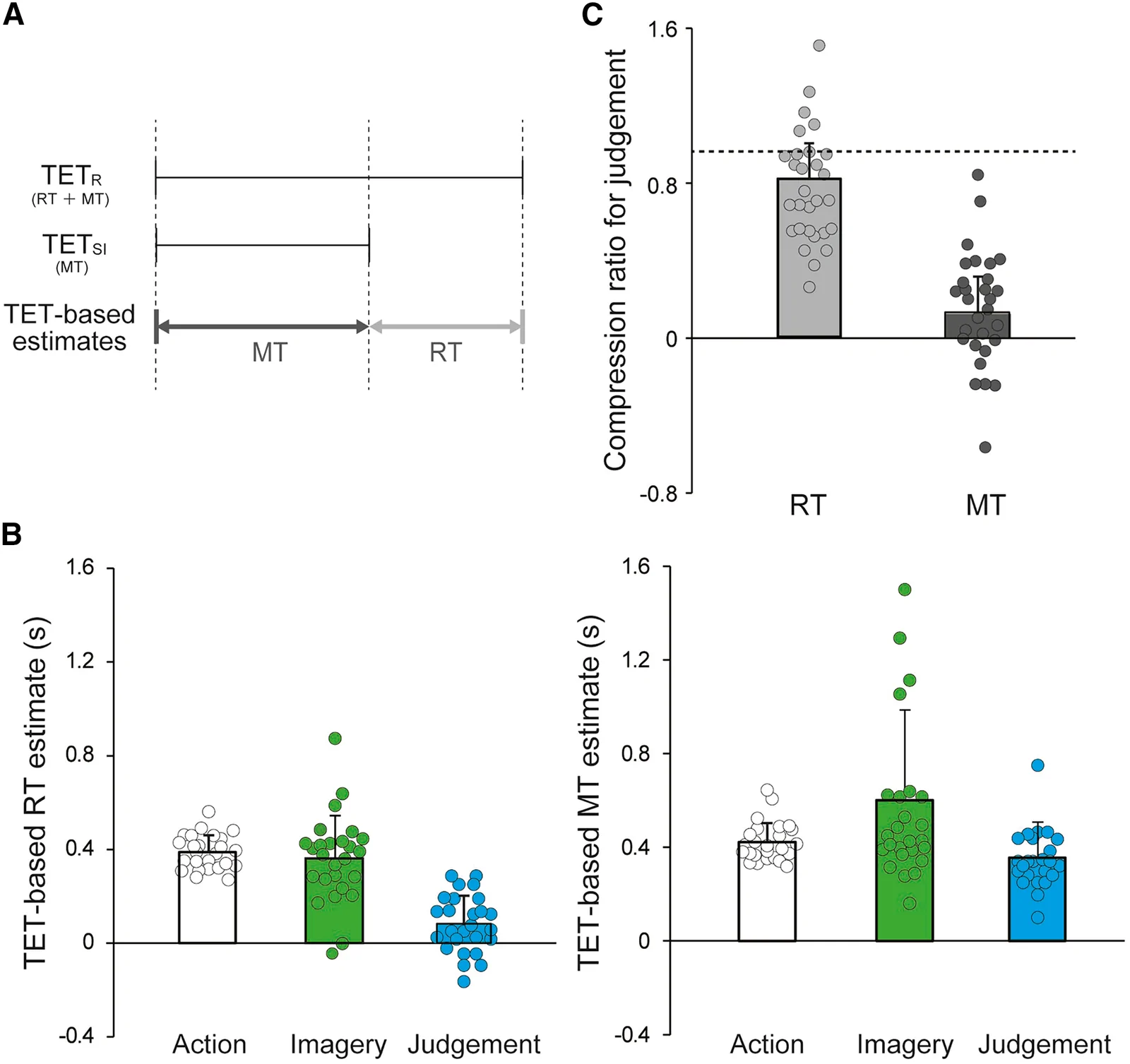
Dissociating RT- and MT-related components and behavioral results of experiment 2 (A) Schematic illustration of the calculation of TET-based estimates. TET_R_ and TET_SI_ represent TETs in the R and SI conditions, respectively. (B) TET-based RT estimate and MT estimate for each task. Each plot and bar shows the mean TET-based RT and MT estimates for each participant and across participants, respectively. Error bars represent standard deviation. (C) Compression ratio of RT and MT for the judgment task. Each plot and bar shows the mean value for each participant and across participants, respectively. A value of 0 indicates equality between the judgment and action components, values of 0–1 indicate relative shortening in the judgment task, and negative values indicate that the judgment component was longer than the corresponding action component. For RT, values >1 occur when TET in the SI condition was longer than that in the R condition, yielding a negative estimated judgment RT. Error bars represent standard deviation.

Importantly, the TET-based RT estimate (0.08 ± 0.12 s) in the judgment task was markedly shorter than in the action and imagery tasks (Figure 3B). To quantify temporal compression during temporal possibility judgments, we calculated a compression ratio for each participant (with a value of 1.0 indicating complete compression; see quantification and statistical analysis). The compression ratio was substantial (0.82 ± 0.29) for RT, whereas it was small (0.15 ± 0.29) for MT (Figure 3C). These results suggest that the overestimation of time-constrained reachability arises primarily from temporal compression of the RT-related component rather than the MT-related component.

### Limited contribution of misperception of target duration to temporal overestimation

Temporal possibility judgment of action would rely not only on the internal representation of the time required for one’s own action, but also on the internally represented duration of the external event (see Figure 1). Specifically, if the target duration was perceived as longer than its actual duration, overestimation of time-constrained reachability would be observed regardless of the compression of movement execution duration assumed above. To assess this possibility, we used a time reproduction task to examine the subjective duration of target presentation during temporal possibility judgment, and its influence on temporal possibility judgment.

Participants (*N* = 12) who had taken part in experiment 1 or 2 performed a time reproduction task, in which they memorized target durations (0.1–1.0 s; orange or yellow) and then reproduced them using a keypad. They then completed one of three conditions: reachability judgment, color judgment, or control (Figure 4A). In the reachability judgment task, as in experiments 1 and 2, participants indicated whether reaching was possible or impossible within the target presentation duration. In the color judgment task, participants responded to the question “Was the target color orange?” using the keypad. In the control condition, no additional task was required. The reachability judgment task was introduced to examine whether subjective time would be selectively prolonged during temporal possibility judgments, given that time perception can be influenced by cognitive states.^21^ To control for the possibility that any observed effects owed merely to the attentional demands of performing a secondary task,^22^ we also included the color judgment task. Each task condition was completed in a block of trials before the next task began. The order of the three tasks was counterbalanced across participants. Before the time reproduction task, each participant performed action and imagery tasks.

**Figure 4:**
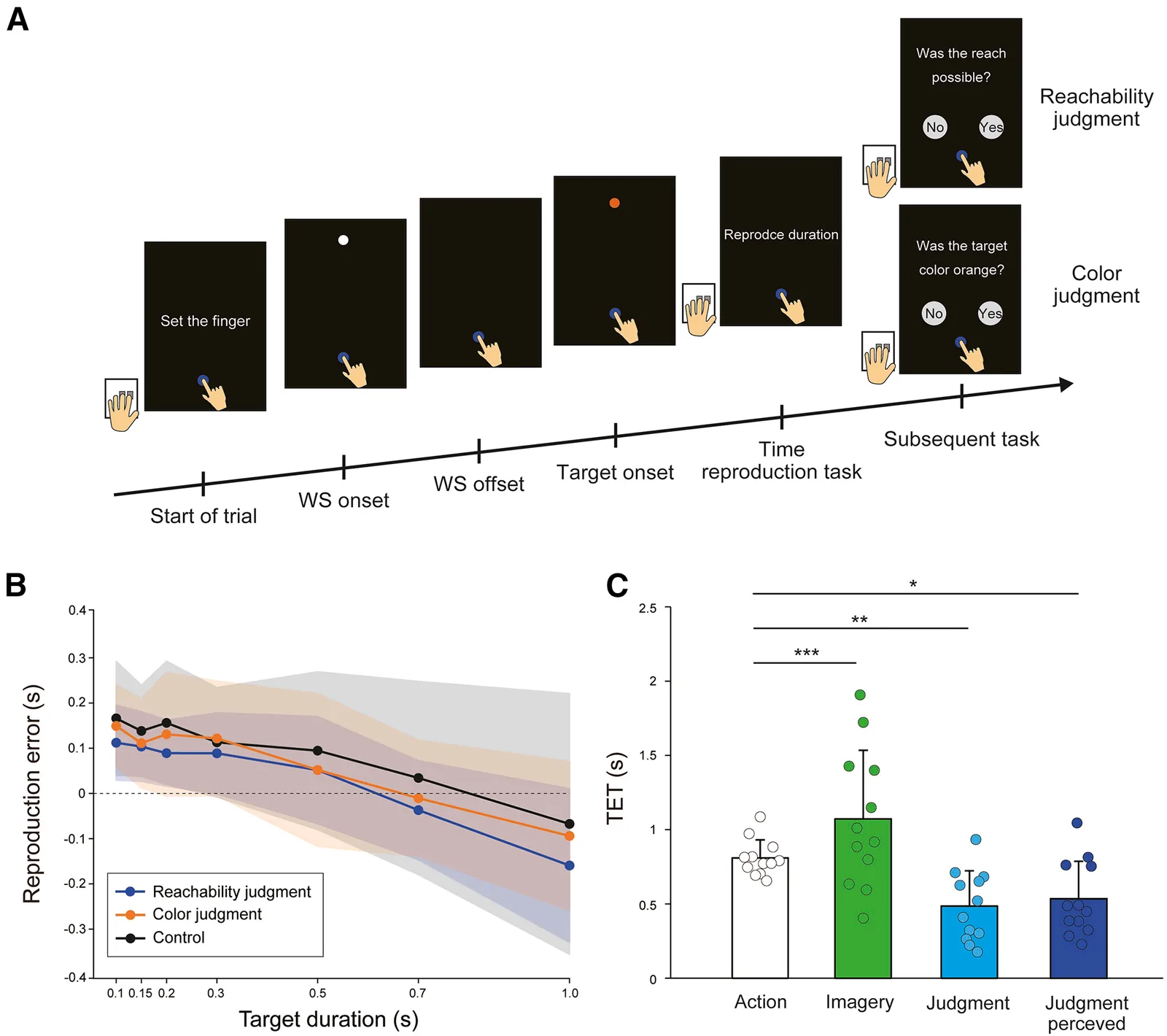
Experimental procedure and behavioral results of experiment 3 (A) Schematic illustration of the trial sequence. Participants first performed a time reproduction task by memorizing and reproducing the duration of a colored target (orange or yellow). After reproducing the duration, they completed the reachability judgment task, color judgment task, or control condition. (B) Group mean reproduction errors as a function of target duration in the three task conditions. Shaded areas indicate ±1 standard deviation. (C) Mean TETs averaged across participants in each condition. Error bars represent ±1 standard deviation of the mean, and dots indicate individual data points. TET, total execution time; WS, warning signal. ∗∗: *p* < 0.01, ∗∗∗: *p* < 0.001 (paired *t* tests with Bonferroni correction).

The reproduced target duration tended to be longer than the actual duration (i.e., positive reproduction error) for short durations (0.1–0.4 s), regardless of the subsequent task (Figure 4B; see also Table S5). To directly evaluate how the perception of target duration influenced the temporal possibility judgment, we fitted the psychometric function (probit function) to the possible/impossible response data as a function of the “reproduced,” but not the actual, target duration (Figure S2), and calculated the PSE at which the fitted function reached 50% of the response as the *judgment-perceived* TET. For the psychometric functions fitted to the actual target durations, the mean R^2^ was 0.98 ± 0.03 and the mean WR was 0.31 ± 0.14 (see Table S6 for individual fitting metrics). One-way repeated-measures ANOVA revealed a significant main effect of Task (*F*_1.67, 18.32_ = 16.05, *p* < 0.001, $\eta_{p}^{2}$ = 0.59). *Post hoc* tests showed that both judgment (0.49 ± 0.24 s) and judgment-perceived TETs (0.54 ± 0.25 s) were significantly shorter than action TET (0.81 ± 0.12 s; judgment, *t*_11_ = 4.50, *p* = 0.005, Cohen’s *d* = 1.30; judgment-perceived, *t*_11_ = 3.40, *p* = 0.04, Cohen’s *d* = 0.98; Figure 4C), and no significant difference between judgment and judgment-perceived TET was found (*t*_11_ = 1.79, *p* = 0.61, Cohen’s *d* = 0.52). These results indicate that although the target duration, especially at short time ranges, was perceived as longer than its actual length, this misperception had only a limited influence on the temporal possibility judgment. Rather, this finding supports our view that the overestimation of time-constrained reachability primarily arises from temporal compression of the RT-related component.

### Behavioral validation of the temporal possibility judgment task

To validate the temporal possibility judgment task in our paradigm, we examined decision time and its relationship to task parameters, as well as the correspondence between judgment and action measures.

We first analyzed *decision time* in the judgment task. Decision time was defined as the interval between the disappearance of the target and the participant’s possible/impossible response. At the single-participant level, decision time tended to be longer for target durations near the PSE (Figure S3). To quantify this relationship across participants, we calculated the absolute temporal difference between the target duration in each trial and the individual PSE (termed the *temporal distance*), and then tested whether decision time was associated with temporal distance on a trial-by-trial basis using a linear mixed-effects model. This analysis revealed a significant negative effect of temporal distance on decision time across all conditions in experiments 1–3 (*p* < 0.001; see Table S7 for details), indicating that decision times increased as the presented duration approached the PSE. This pattern suggests that the judgment became more difficult near the boundary between “possible” and “impossible” responses, consistent with a comparison process between the presented duration and an internal judgment criterion.

Next, we present two results linking judgment measures to action-related processes. First, as reported above (see “Overestimation of time-constrained reachability”), TET in the judgment task was longer for far targets than for near targets (Figure 2C), showing a systematic effect of movement distance. A similar pattern was observed in the action task. Second, we examined the relationship between action and judgment components across participants using data from experiments 1 and 2, and control experiment (*N* = 47; see quantification and statistical analysis). We found a significant positive effect of action TET on judgment TET, independent of target location and condition (Table S8; Figure S4), indicating that longer action TET was associated with longer judgment TET. These results suggest that participants referred to the expected duration of the actual action, while taking into account individual motor properties and action goals when performing the temporal possibility judgment task.

## Discussion

Using a novel experimental paradigm, we showed robust overestimation of time-constrained reachability, a phenomenon in which participants judged a target as reachable even when the available time was significantly shorter than the actual execution time (experiments 1–3). Crucially, we found that this overestimation is primarily attributable to a marked compression of the RT-related component rather than inaccurate estimation of the MT-related component (experiment 2). Furthermore, we confirmed that this bias is not attributable to the misperception of the target duration as an external event (experiment 3).

In the judgment task, TET increased with movement distance (Figure 2C), mirroring the findings of the action task. This distance-dependent pattern suggests that participants implicitly simulated the reaching movement when temporally judging reachability, supporting the view that prospective action judgments involve covert motor simulation.^15^^,^^16^^,^^17^^,^^23^ However, the overall level of TET differed between the two cognitive tasks: imagery TET was longer than action TET, consistent with previous studies,^9^^,^^10^^,^^11^^,^^12^^,^^13^^,^^14^ whereas judgment TET was shorter. This divergent pattern between the two cognitive tasks indicates that the implicit representation of reaching movement underlying temporal possibility judgments may differ qualitatively from the explicit representation engaged in the imagery task. This interpretation aligns with Jeannerod’s framework,^18^^,^^19^ which distinguishes overt (explicit) and covert (implicit) mental simulations of action according to the presence of conscious experience accompanying the task. In fact, several studies have reported inconsistent results between implicit and explicit motor imagery in tasks specifically requiring each type of processing.^24^^,^^25^^,^^26^

The most striking finding of this study is that temporal compression was observed in the RT-related component, but not in the MT-related component (Figure 3C). The RT-related component reflects multiple processes preceding movement onset, including perceptual encoding, decision-making, motor preparation, and early motor command specification, whereas the MT-related component is more closely associated with movement execution, including continuous motor command generation and sensory feedback processing.^27^ Our results therefore suggest that covert simulation during temporal possibility judgment may represent processes contributing to movement execution more faithfully than those contributing to the RT-related component. Motor imagery is thought to rely on a forward model that is also engaged during action planning.^28^^,^^29^ The forward model is proposed to continuously predict and update the sensory consequences of efferent motor commands during movement execution.^30^^,^^31^^,^^32^ Together, these considerations suggest that temporal possibility judgments may incorporate processes occurring during MT, possibly because ongoing movement-related processing can be internally simulated through forward-model mechanisms. By contrast, some processes preceding overt movement onset may not be fully simulated and may therefore contribute less to temporal possibility judgments.

We must also consider the possibility that the observed overestimation of time-constrained reachability is attributable to a bias in the decision-making process, because the temporal possibility judgment ultimately involves comparing one’s estimated movement duration with the target presentation time (Figure 1). In the absence of penalties for incorrect judgments, as in the present study, participants can adopt a liberal criterion^33^^,^^34^ favoring “possible” responses to maximize success chances, potentially leading to the overestimation of reachability. However, if this decision bias were primarily responsible, similar overestimation would be expected in the SI condition (which required no motor response) as well as in the R condition (experiment 2), because such a bias would operate at the decision stage, independent of motor response requirements. We therefore believe that the substantially smaller overestimation in the SI condition (Table 1) makes a general decision bias alone unlikely to account for the observed effect and instead supports the involvement of a distortion in the motor simulation process, specifically linked to the RT-related component. Nevertheless, we cannot exclude the possibility that the response criterion differed between the R and SI conditions, with the R condition promoting a stronger tendency to respond “possible.” Hence, a context-dependent decision bias may have contributed, at least in part, to the observed overestimation of time-constrained reachability.

In conclusion, this study demonstrates a novel and robust cognitive bias in temporal possibility judgment of action. We found that the duration associated with the RT-related component was selectively underestimated during temporal possibility judgments, whereas the MT-related component was estimated relatively accurately. These findings provide new insight into how humans evaluate the temporal possibility of their own actions and suggest that the present psychophysical paradigm offers a useful framework for examining different temporal components of action estimation. Premotor cortical populations have been shown to encode temporal and contextual information relevant to action and decision-making^35^^,^^36^; however, the present behavioral findings do not allow us to determine whether these neural populations contribute to the RT-related compression observed here. Future studies combining this paradigm with neurophysiological measurements will be needed to clarify the neural mechanisms underlying this temporal compression.

### Limitations of the study

There were several limitations to our study. First, the RT- and MT-related measures in experiment 2 were operational estimates based on the assumption that movement execution was comparable between the R and SI conditions. Given that urgency or movement strategy could have differed across conditions, the R–SI difference may partly reflect differences in movement execution component. Second, the sample was imbalanced in sex and age, with few female participants (experiments 1 and 3) and a predominance of individuals in their early twenties. Third, in experiment 1, task order was not fully counterbalanced because imagery always preceded judgment. Although the control experiment replicated the main finding without prior imagery, other order effects cannot be completely excluded. Fourth, the relatively small samples and absence of an *a priori* power analysis may have limited the detection of small effects and interactions. Finally, because the study used rapid, visually guided reaching movements, the findings may not generalize to other sensory modalities (e.g., audition) or to other action contexts, such as whole-body actions, longer-duration movements, or decisions involving explicit costs.

## Resource availability

### Lead contact

Requests for further information and resources should be directed to and will be fulfilled by the lead contact, Keisuke Tani (keisuketani.pt@gmail.com).

### Materials availability

This study did not generate new, unique reagents.

### Data and code availability

• Participant-level behavioral data and all original code used in this study are publicly available at https://doi.org/10.17605/OSF.IO/M823Y. For the action task, processed measures, including reaction time (RT) and movement time (MT), are provided, whereas the original frame-by-frame kinematic data are not publicly available because of potential privacy concerns.
• Any additional information required to reanalyze the data reported in this study is available from the lead contact upon request.

## Acknowledgments

This work was supported by internal research funding from Otemon Gakuin University (to K.T. and S.H.) and 10.13039/501100001691JSPS 10.13039/501100001691KAKENHI (22K11654 and 25K14766 to K.K.).

## Author contributions

Conceptualization, K.T., S.O., and K.K.; methodology, K.T., S.H., and K.K.; investigation, K.T. and S.H.; writing – original draft, K.T. and S.H.; writing – review and editing, K.T., S.O., S.H., and K.K.; funding acquisition, K.T., S.H., and K.K.; resources, K.T., S.H., and K.K.; supervision, S.H. and K.K.

## Declaration of interests

The authors declare no competing interests.

## STAR★Methods

### Key resources table

**Table:** 

REAGENT or RESOURCE	SOURCE	IDENTIFIER
**Deposited data**		

Behavioral data from this study	This paper	https://doi.org/10.17605/OSF.IO/M823Y

**Software and algorithms**		

MATLAB 2023b	Math Works	RRID: SCR_001622
Statistics and Machine Learning Toolbox	Math Works	https://www.mathworks.com/products/statistics.html
R, version 4.3.3	R Foundation for Statistical Computing	https://www.r-project.org/
RStudio, version 2023.12.1.402	Posit Software, PBC	https://posit.co/products/open-source/rstudio/
lme4 R package, version 1.1.35.2	CRAN	https://cran.r-project.org/package=lme4
lmerTest R package, version 3.1.3	CRAN	https://cran.r-project.org/package=lmerTest
PsychoPy 2024.2.4	Open Science Tools Ltd.	RRID: SCR_006571
Psignifit 4 toolbox	Schütt et al.^37^	https://github.com/wichmann-lab/psignifit
JASP 0.18.1.0	JASP Team	https://jasp-stats.org/
Adobe Illustrator 2026	Adobe	https://www.adobe.com/products/illustrator.html
Custom analysis code	This paper	https://doi.org/10.17605/OSF.IO/M823Y

### Experimental model and study participant details

#### Participants

In total, 14 healthy volunteers (3 females; mean ± standard deviation age = 22.9 ± 1.5 years) participated in Experiment 1, 26 (11 females; age = 21.5 ± 3.1 years) in Experiment 2, and 12 (no females; age = 22.8 ± 2.5 years) in Experiment 3. In the control experiment, 7 naive healthy volunteers (3 females; age = 21.1 ± 0.4 years) participated. Participants self-reported whether they were male or female. Different participants took part in Experiments 1 and 2, whereas participants in Experiment 3 were recruited from among those who had participated in Experiment 1 or 2. All participants were recruited through poster advertisements and direct invitations within the university community. No *a priori* power analysis was conducted, and sample sizes varied across experiments according to study design and recruitment procedures. All participants were classified as right-handed according to the Flinders Handedness Survey (FLANDERS),^38^ had normal vision, and had no history of neurological disorders. Participants were compensated at a rate of 1,000 Japanese yen per hour. Written informed consent was obtained from each participant before the experiment. This study was approved by the local Ethics Committee of the Faculty of Human and Environmental Studies, Kyoto University (for Experiments 1–3; approval number: 24-H-19) and by the Ethics Committee of the Faculty of Psychology, Otemon Gakuin University (for Experiment 2 and the control experiment; approval number: 2025–56 and 2025–77). The study was conducted in accordance with the Declaration of Helsinki (2013).

#### Apparatus

Participants sat on a chair in front of a table on which a touch display (size, 34.5 cm × 19.5 cm; LCD-CF161XDB-MT; I-O DATA, Kanazawa, Japan) was firmly positioned and aligned with the participant’s body midline. To precisely detect finger position during the action task, participants wore a touch display-compatible finger sleeve on the right index finger, and the finger position data were sampled at 60 Hz. In each task (the action task under the SI condition, imagery, and judgment tasks), participants used their left hand to operate a keypad (TK-TCM011SV; Elecom, Osaka, Japan), which was customized to have only two (Experiments 1 and 3) or three buttons (Experiment 2). The lights in the experimental room were turned off during the tasks. Visual stimuli presentation and participant response acquisition were performed using Psychopy software (version 2024.2.4; Open Science Tools, Nottingham, UK) installed on a PC.

### Method details

#### Task and procedure

##### Experiment 1

Each participant successfully completed the three experimental tasks (action, imagery, and judgment), performing each in a separate block before proceeding to the next. In the action task, participants reached toward a visual target, whereas in the imagery task they imagined performing the reach and indicated the onset and offset of the imagined movement. In the judgment task, participants judged whether the target could be reached within varied presentation durations. The task order was counterbalanced across participants, who were assigned equally to action-first (action → imagery → judgment) and cognitive-first (imagery → judgment → action) groups. Task order was included as a secondary factor to examine whether prior execution of the reaching movement influenced subsequent performance in the imagery and judgment tasks, particularly given previous evidence that actual execution shortens the duration of subsequently imagined actions.^11^^,^^13^^,^^14^^,^^20^ Because the tasks were performed in separate blocks, distance effects were tested within each task and were not interpreted as causal effects across tasks.

Figure 2 shows the trial-wise procedure in each task. For the action task, the participants first put the tip of the right index finger on the blue-colored circle (diameter: 1.0 cm; home position). A white-colored WS (diameter: 1.5 cm) was presented for 1 s at either the near position (distance from the home position: 11.7 cm) or far position (distance from the home position: 23.3 cm). An orange-colored target (diameter: 1.5 cm) was then presented for 2 s at identical positions with a random delay (0, 0.1, or 0.3 s). Participants were instructed to quickly react to the onset of the target and reach it with their right hand as quickly and accurately as possible. After holding the finger on the target for approximately 1 s, participants returned it to the home position, and the next trial then began. To record temporal changes in finger position, participants were asked to keep a finger on the display during movements. The target duration was 4 s, and the interval between the target offset and WS onset in the next trial was 2 s.

For the imagery task, the trial sequence was identical to that of the action task, except that participants imagined reaching the target instead of actually moving. They held down the right key with their left index finger while waiting for target onset. Upon target presentation, they indicated the onset of their imagined movement by releasing the right key. The offset of the imagined movement was indicated by pressing the left key with their middle finger, which simultaneously triggered the disappearance of the target. The interval between the target offset and WS onset of the next trial was set at 2 s.

For the judgment task, the target was presented for a specific duration at either the near or far position after the WS disappeared. The target durations were 0.1, 0.15, 0.2, 0.3, 0.5, 0.7, and 1.0 s, presented in a randomized order. After the target disappeared, the text “Was the reach possible within the time?” was presented, and participants answered this question using the keypad. In both the imagery and judgment tasks, participants were asked to keep their right index finger at the home position.

Each participant performed 20 trials (10 at each target location) in the action and imagery tasks, and 140 trials (10 for each target duration and location) in the judgment task. The judgment task trials were divided into two blocks with a 5-min break between them. For practice, before the main trials, each participant completed a few trials of the action and judgment tasks, whereas in the imagery task they repeated the trials until they felt confident in accurately imagining the movement and indicating its timing using the keypad. Only the cognitive-first group performed a preliminary reaching task before practice trials for the imagery task, in which they sequentially reached toward targets presented on the display. The purpose of this task was to familiarize them with the level of finger friction when sliding across the display surface. A break of approximately 2 min was provided between tasks. The action and imagery tasks each took approximately 15 min, whereas the judgment task took approximately 30 min.

##### Experiment 2

Participants completed the action, imagery, and judgment tasks as in Experiment 1, but each task was performed under two conditions: R and SI. In the R condition, participants reacted to target onset and reached toward the target (same as Experiment 1), whereas in the SI condition, participants reached toward the target according to a self-determined timing. The task order was the same for all participants (action → imagery → judgment), whereas the order of conditions within each task was counterbalanced by assigning participants equally to two groups: R-first (R → SI) and SI-first (SI → R). Only the far position was used for the target location in this experiment.

In the R condition, as in Experiment 1, the target appeared at a random delay after the WS. By contrast, in the SI condition, the timing of target onset was determined by the participants themselves. Specifically, participants pressed and held the left key at the beginning of each trial and, after the WS appeared, initiated target presentation by releasing the key. This approach eliminated the temporal uncertainty of target onset, allowing participants to judge target reachability without needing to account for the time required for reaction. After target onset, the procedure in both the R and SI conditions was the same as in Experiment 1. In the judgment task, the target presentation durations consisted of eight conditions, identical to those in Experiment 1, with the addition of a 0.4 s condition.

Each participant performed 20 trials (10 for each condition) in the action and imagery tasks, respectively, and 160 trials (10 for each target duration and condition) in the judgment task. Judgment trials for each condition were split into two blocks with a 5-min break. As in Experiment 1, participants performed a few practice trials in the action and judgment tasks, and repeated the imagery task until they felt confident in indicating its timing. The action and imagery tasks each took approximately 20 min, whereas the judgment task took approximately 40 min.

##### Experiment 3

In this experiment, we examined whether the bias in temporal possibility judgment reflected distorted perception of the target duration rather than action-related simulation. Participants reproduced each target duration, and reachability judgments were analyzed according to both the reproduced and actual duration.

Participants performed the time reproduction task following completion of the action and imagery tasks, as in Experiments 1 and 2. At the beginning of each trial, they pressed the right button of the keypad with their left index finger. After the WS disappeared, a target colored either orange or yellow was presented for a specific duration with a random delay (0, 0.1, or 0.3 s). Participants were then asked to reproduce the duration of the target presentation by releasing the right button and pressing the left button with the left middle finger. Across trials, target durations were randomly selected from the seven durations used in the judgment task in Experiment 1.

After each reproduction trial, participants completed the reachability judgment task, the color judgment task, or a control condition. In the reachability judgment task, participants indicated whether reaching the target within the target presentation time was possible. In the color judgment task, they indicated whether the target color was orange using the keypad. No response was required in the control condition. Each task condition was completed as a block of trials before moving on to the next task, and the order of the three tasks was counterbalanced across participants.

Each participant completed 10 trials for the action and imagery tasks, and 56 trials for each subsequent task condition (i.e., 168 trials in total) for the time reproduction task. As in Experiments 1 and 2, participants practiced a few trials before the action and time reproduction tasks, and repeated the imagery task until they felt confident in indicating its timing. A short break of approximately 2 min was provided between tasks. The action and imagery tasks each took approximately 10 min, whereas time reproduction task took approximately 45 min (15 min for each condition).

#### Control experiment

We conducted a control experiment to confirm that the reduction in judgment TET relative to observed action TET was not attributable to the imagery task preceding the judgment task. Participants naive to Experiments 1–3 performed the judgment task without an imagery task, followed by the action task. Only the far position was used, and the target duration was identical to that in Experiment 1. As in Experiment 1 (cognitive–first group), participants performed a preliminary reaching task before the judgment task to familiarize themselves with the level of finger friction on the display surface. Each participant completed 70 trials (10 trials each for seven target durations) in the judgment task and 10 trials in the action task.

### Quantification and statistical analysis

#### Rejection of trials

In each experiment, trials in which no response was obtained from the keypad or touch display within the allotted time after target onset (action, 2 s; imagery/judgment, 5 s) were excluded. Trials in which a response could not be properly recorded were also excluded from the analyses. As a result, 64 trials (1.12% of all trials) were removed for Experiments 1–3. In Experiment 2, because none of the action task trials in the R condition were properly recorded for one participant (ID: 3), that participant’s data were excluded from the corresponding statistical analyses.

#### Data analysis

##### Temporal performance of each task

For the action and imagery tasks, RT was defined as the interval between target onset and movement onset, and MT as the interval between movement onset and movement offset. TET was defined as the sum of RT and MT:

TET=RT+MT

For the *action* task, the finger position on the display was recorded at a nominal sampling rate of 60 Hz. However, the actual frame intervals were slightly irregular. To obtain a uniformly sampled time series, the finger position data were resampled at 120 Hz using the *resample* function of MATLAB (version 2023b; MathWorks, Natick, MA, USA). Subsequently, a low-pass filter with a second-order Butterworth filter (cutoff frequency = 10 Hz) was applied to the data for kinematic analyses. Movement onset was defined as the first time point at which the velocity exceeded 10 cm/s, and movement offset was defined as the first time point at which the velocity dropped below 10 cm/s while the finger position was within the target area.^39^

For the *imagery* task, movement onset and offset were the time points at which the right button of the keypad was released and the left button was subsequently pressed, respectively. Imagery RT and MT were calculated using these reported onset and offset times, and imagery TET was calculated as their sum. In the SI condition of Experiment 2, participants initiated the action or imagined action without an externally presented target-onset cue. Therefore, RT was not defined for either the action or imagery task, and TET corresponded to MT.

For the *judgment* task, we fitted the psychometric function (cumulative Gaussian function) to the proportion of the possible responses at each target duration for each participant and task condition. The PSE, defined as the target duration corresponding to a 50% probability of a “possible” response, was used as the judgment TET. Psychometric functions were fitted using the Psignifit 4 toolbox^37^ in MATLAB. Detailed fitting metrics for the psychometric functions, including PSE confidence intervals, slope, lapse rate, Weber ratio, and goodness-of-fit indices (deviance, R^2^), are reported in Tables S1, S3, and S6. No participants were excluded on the basis of poor psychometric fits or extreme PSE estimates.

In Experiment 2, TET-based RT and MT estimates were derived for all three tasks using the same operational decomposition. The *TET-based MT estimate* was defined as the TET in the SI condition, whereas the *TET-based RT estimate* was defined as the difference between the TETs in the R and SI conditions (see Figure 3A). For the action and imagery tasks, directly measured RT and MT were also available and were used to assess the validity of this decomposition. By contrast, RT and MT could not be measured directly in the judgment task. Accordingly, the TET-based RT and MT estimates for the judgment task should be regarded as operational estimates rather than as direct or process-pure measures of distinct RT and MT processes.

In Experiment 3, we also examined reachability judgments according to the reproduced, not the actual, target duration, given that participants’ internally represented durations could deviate from the actual target durations. The reproduced durations were generally unique and therefore could not be meaningfully aggregated into response proportions at fixed durations. Accordingly, we fitted the trial-by-trial binary response data using a binomial generalized linear model with a probit link, implemented with the *fitglm* function in MATLAB. This approach, like the cumulative Gaussian fit used in Experiments 1 and 2, models response probability using the cumulative standard normal function. The PSE, defined as the reproduced time at which the fitted probit function predicted a 0.5 probability of a “reachable” response, was calculated as the judgment-perceived TET.

##### Compression ratio of RT- and MT-related components during temporal possibility judgment

In Experiment 2, using the TET data in the R (including RT and MT) and SI conditions (including only MT), the RT for each task was calculated by subtracting the TET in the SI condition from that in the R condition, and MT was defined as the TET in the SI condition. Then, to quantify the extent of RT and MT compression during possibility judgment, we computed the compression ratio separately for RT and for MT for each participant:

$$Compression\;ratio=1-\frac{Judgement}{Action}$$

A compression rate of 0 indicates no compression, whereas values approaching 1 indicate progressively greater compression of the judgment component relative to the corresponding action component; a value of 1 indicates complete compression. Negative values indicate that the judgment component was longer than the corresponding action component (i.e., expansion of judged time). For RT, values >1 can occur when the judgment TET in the SI condition exceeds that in the R condition, resulting in a negative estimated judgment RT. Such values indicate a reversal of the expected difference between the R and SI conditions rather than compression exceeding 100%.

##### Time reproduction performance

For the time reproduction task in Experiment 3, the reproduced time was computed for each trial as the time between the time point when the right button was released and that when the left button was pressed. Using the reproduced time data, we calculated the reproduction error by subtracting the duration of target presentation from the reproduced time. A positive reproduction error indicates overestimation of the target duration.

#### Statistical analysis

To compare TET across tasks, we performed three-way mixed-design ANOVAs with within-participant factors of Task (action/imagery/judgment) and either Distance (Near/Far, Experiment 1) or Condition (R/SI, Experiment 2), and a between-participant factor of Group (action-first/cognitive-first in Experiment 1; R-first/SI-first in Experiment 2). For Experiment 3, we performed one-way repeated-measures ANOVA with a within-participant factor of Task (action/imagery/judgment/judgment-perceived). Prior to ANOVAs, Mauchly sphericity tests were performed. When sphericity was violated, Greenhouse–Geisser-corrected degrees of freedom and *p* values were reported. For each analysis, when significant main or interaction effects were detected, simple main effects were examined, followed by Bonferroni-corrected post hoc pairwise comparisons where appropriate. Statistical significance in figures is indicated by asterisks (∗*p* < 0.05, ∗∗*p* < 0.01, and ∗∗∗*p* < 0.001), based on Bonferroni-corrected post hoc comparisons. These analyses were conducted using the Statistics and Machine Learning Toolbox for MATLAB. Partial eta squared ($\eta_{p}^{2}$) and Cohen’s *d* were reported as measures of effect size for ANOVAs and post hoc multiple comparisons, respectively.

To assess the relationship between TETs across the action and judgment tasks, we performed a linear mixed-effects model analysis using the data (*N* = 47) from Experiments 1 and 2, and the control experiment. The model was specified as follows:

lmer(judgmentTET∼actionTET∗Targetdistance+actionTET∗Condition

+(1|participant))

As fixed effects, action TET, Target distance (far vs. near), and Condition (R vs. SI) were included along with their interaction, and random intercepts were included for participants.

In Experiment 3, to compare the reproduction errors across task conditions, we performed two-way repeated-measures ANOVA with factors of Duration (0.1–1.0 s) and Task condition (reachability judgment/color judgment/control). In addition, we examined whether the reproduction errors deviated from zero (i.e., no error) using Bayesian one-sample t-tests. Bayes factors (*BF*_10_) for each stimulus duration and task condition were calculated with JASP (version 0.18.1.0; JASP Team, Amsterdam, the Netherlands) using the default Cauchy prior with scale parameter *r* = 0.707. We interpreted *BF*_10_ > 3 as evidence for the alternative hypothesis and *BF*_10_ < 1/3 as evidence for the null hypothesis, with values between 1/3 and 3 considered anecdotal/insensitive evidence.^40^
