# Supplementary material for: Reaction time-related compression underlies overestimation of time-constrained reachability
Source: iScience. 2026 Oct 31;29(11):118115. doi: 10.1016/j.isci.2026.118115 (PMC13743933; doi:10.1016/j.isci.2026.118115)
Supplement: Supplementary file 1 [file mmc1.pdf]

iScience, Volume *29*

Supplemental information

Reaction time-related compression underlies overestimation of time-constrained reachability

Keisuke Tani, Soma Okuuchi, Satoshi Hirose, and Keisuke Kushiro

Figure S1. Results of the control experiment.

This experiment was performed to test whether the temporal compression of judgment total execution time (TET) occurred even when the imagery task was not performed before the judgment task. A paired t-test showed that judgment TET (0.36 ± 0.10 s) was significantly shorter than action TET (0.68 ± 0.11 s; $t_6 = 6.53$, $p < 0.001$, Cohen's $d = 2.47$). Error bars represent ± 1 standard deviation of the mean, and dots indicate individual data points.

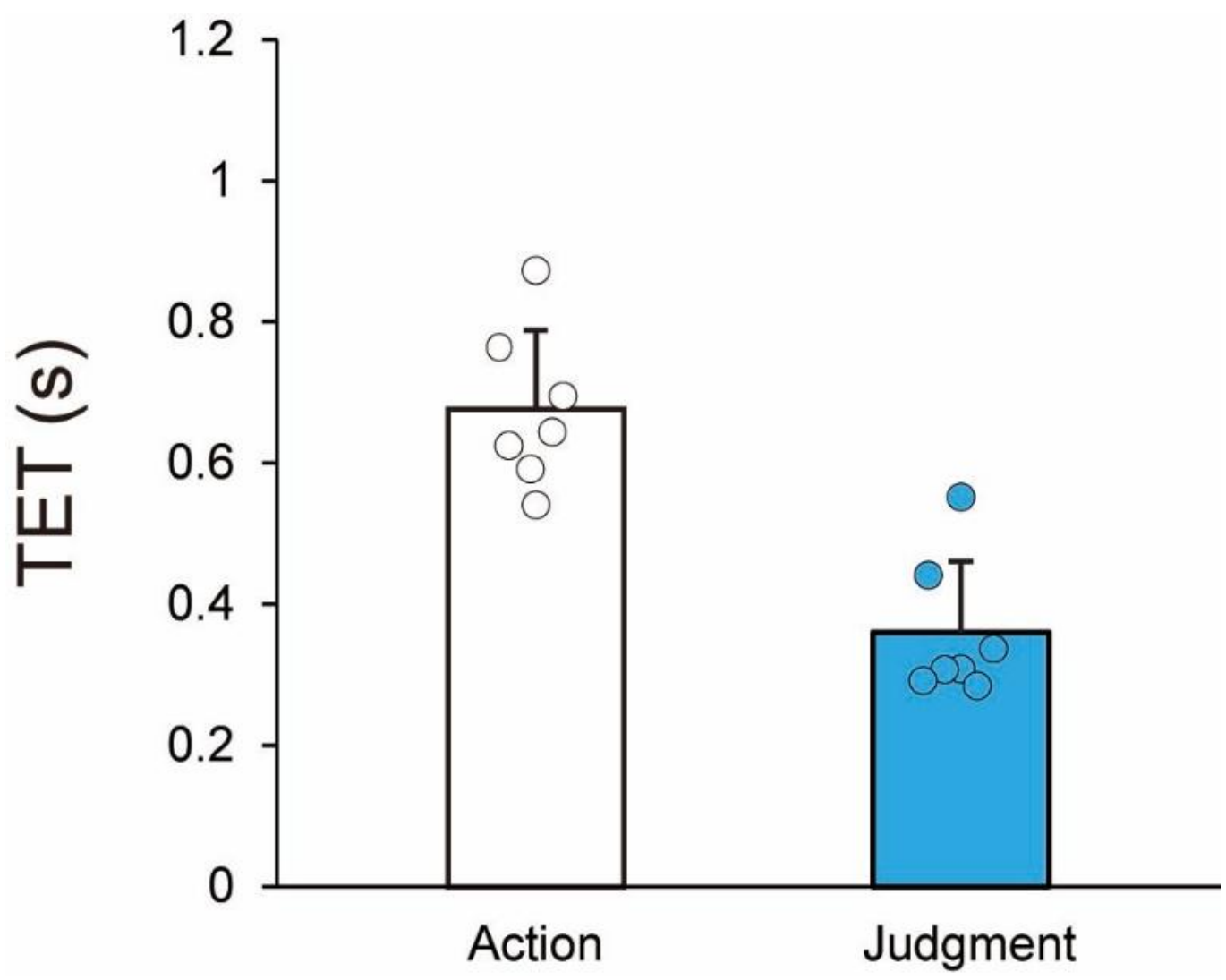

Figure S2. Psychometric functions fitted to the trial-by-trial responses as a function of the reproduced target duration for each participant in the reachability judgment task of Experiment 3.

0 and 1 indicate "impossible" and "possible" responses, respectively. Grey circles indicate individual responses, and solid black lines represent fitted probit functions. The intersection of each psychometric function with the horizontal grey dotted line (response proportion = 0.5) indicates the perceived-judgment total execution time, shown at the bottom of each vertical line.

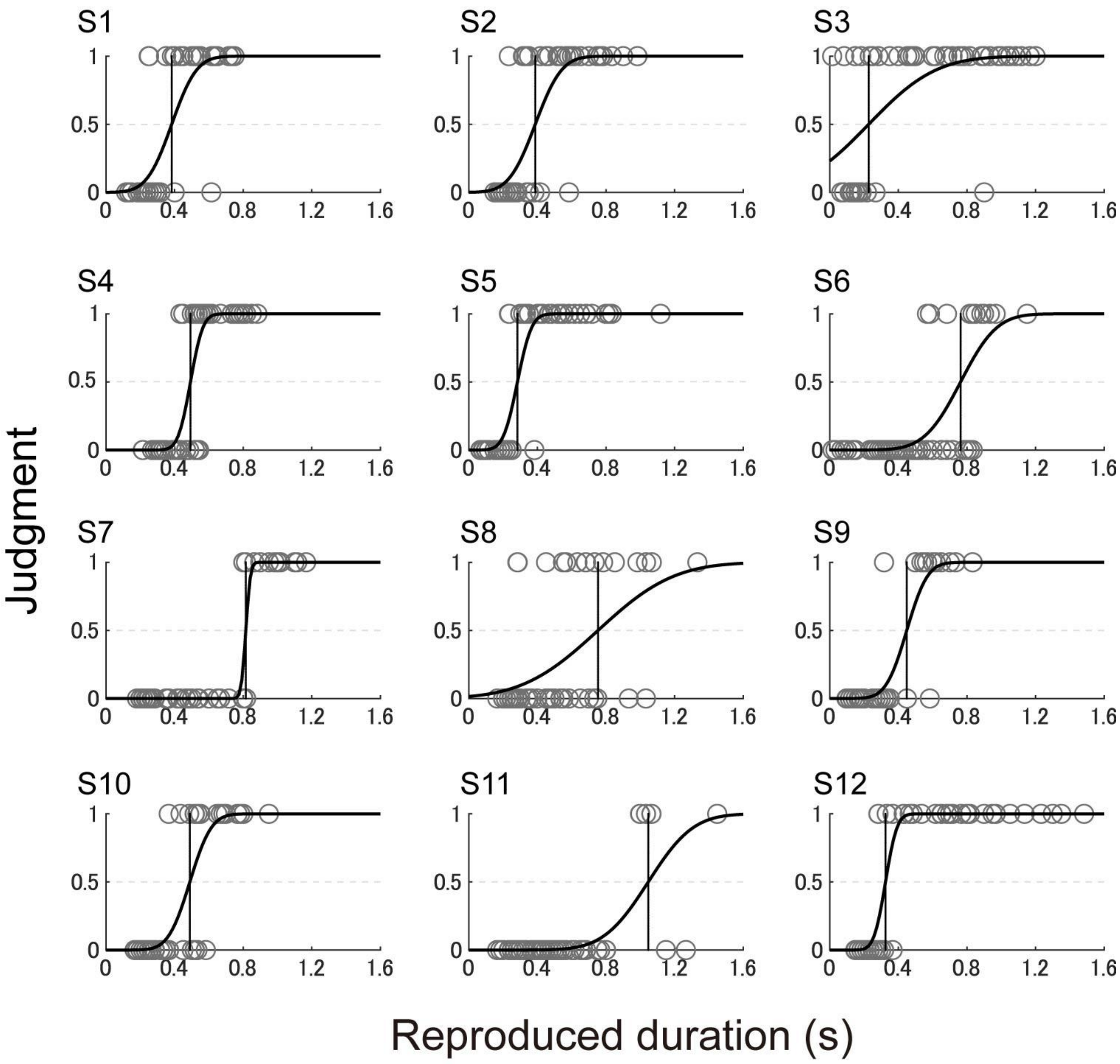

Figure S3. Decision time patterns in a representative participant (ID = 1 in Experiment 1 for the far target).

Decision time as a function of target duration. The dot line represents target duration corresponding to the PSE (judgment TET).

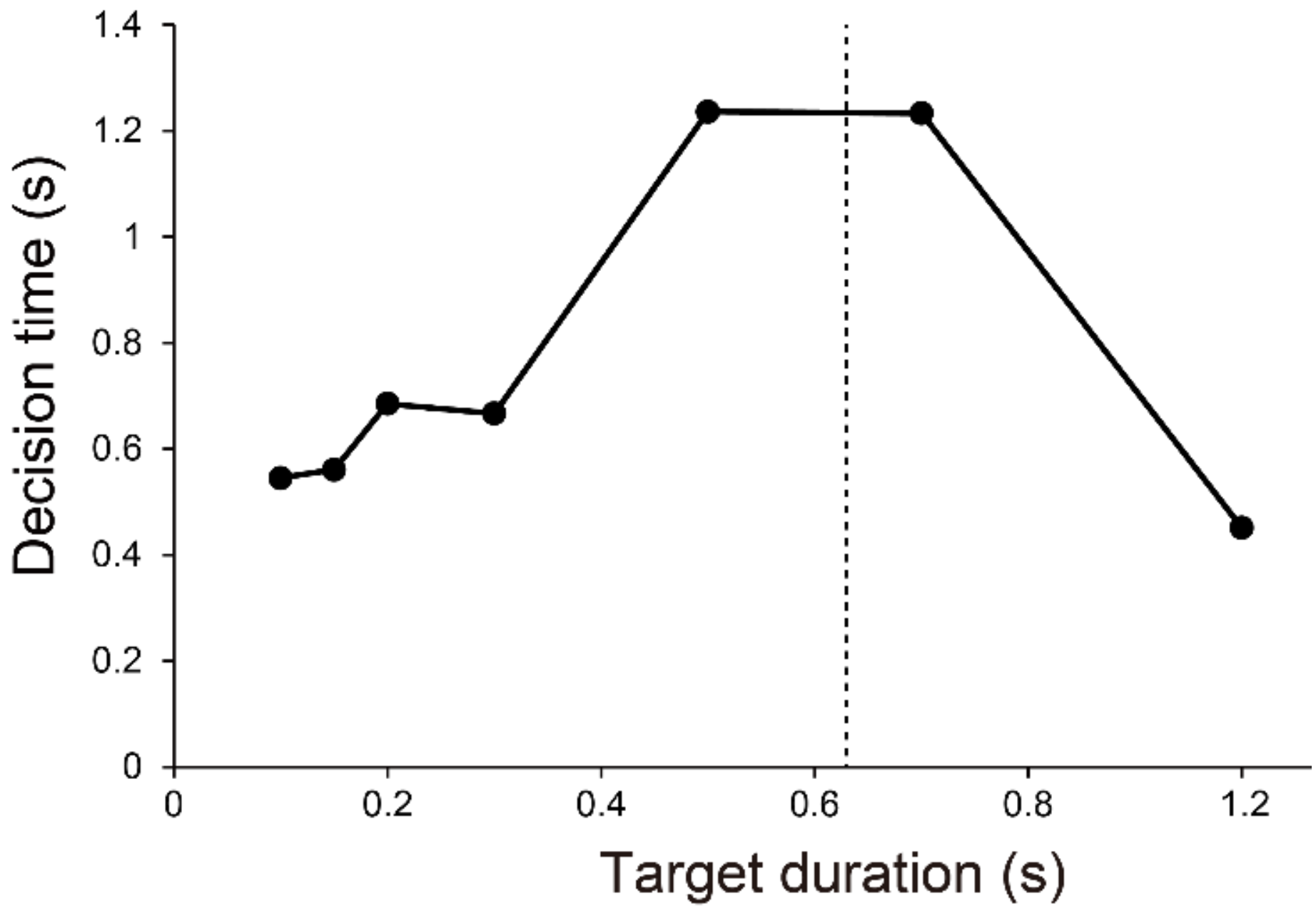

Figure S4. The relationship between action and judgment TETs.

Each plot shows the individual data from Experiments 1 and 2, and the control experiment. The dotted line represents the identity line (judgment TET / action TET = 1).

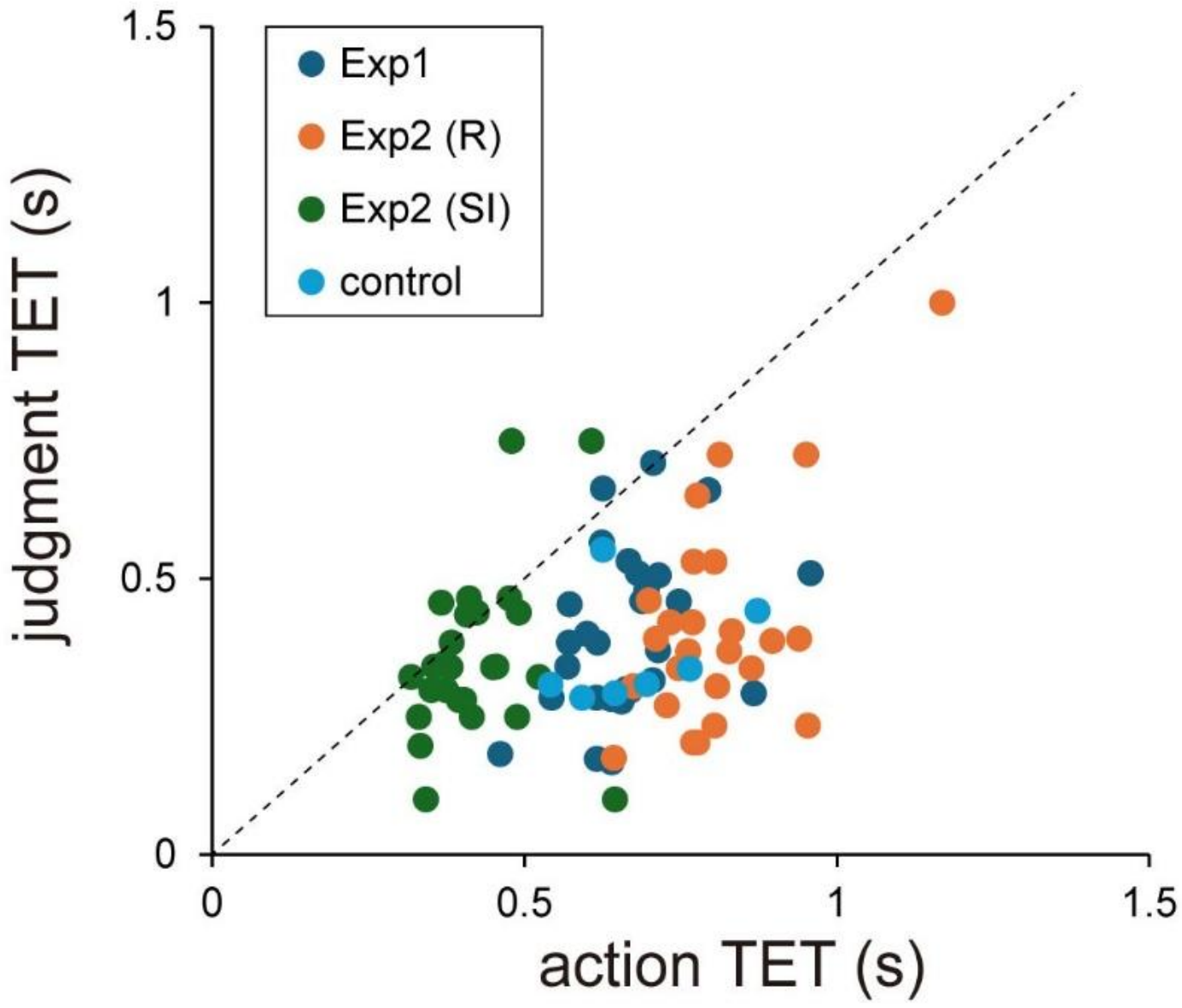

Table S1. Psychometric function fitting metrics for the judgment task in Experiment 1.

Sub ID	Target distance	PSE	95% CI	slope	lapse	WR	deviance	R^2
1	Near	0.57	0.46–0.67	0.44	0.00	0.32	1.05	0.99
	Far	0.71	0.61–0.85	0.45	0.00	0.26	0.78	0.99
2	Near	0.29	0.25–0.37	0.10	0.00	0.14	2.33	1.00
	Far	0.51	0.43–0.72	0.12	0.14	0.12	0.58	0.99
3	Near	0.28	0.24–0.36	0.22	0.00	0.33	1.17	0.99
	Far	0.51	0.41–0.61	0.46	0.00	0.37	0.61	1.00
4	Near	0.30	0.26–0.38	0.22	0.00	0.30	0.44	1.00
	Far	0.46	0.33–0.56	0.48	0.00	0.43	2.32	0.99
5	Near	0.38	0.32–0.48	0.33	0.00	0.35	1.98	0.99
	Far	0.53	0.40–0.63	0.46	0.00	0.35	3.33	0.96
6	Near	0.38	0.31–0.47	0.28	0.00	0.30	0.50	1.00
	Far	0.51	0.45–0.60	0.13	0.00	0.11	0.00	1.00
7	Near	0.28	0.25–0.36	0.19	0.00	0.28	0.32	1.00
	Far	0.37	0.30–0.46	0.29	0.00	0.33	1.22	0.99
8	Near	0.17	0.11–0.23	0.20	0.00	0.49	1.41	0.97
	Far	0.32	0.29–0.43	0.10	0.03	0.13	2.33	1.00
9	Near	0.17	0.11–0.25	0.25	0.00	0.59	3.61	0.93
	Far	0.30	0.27–0.39	0.10	0.03	0.14	2.34	1.00
10	Near	0.45	0.35–0.49	0.12	0.00	0.11	0.00	1.00
	Far	0.66	0.57–0.78	0.40	0.00	0.25	0.11	1.00
11	Near	0.18	0.16–0.24	0.14	0.00	0.31	1.07	0.99
	Far	0.34	0.30–0.44	0.26	0.00	0.31	1.92	0.99
12	Near	0.28	0.25–0.35	0.10	0.00	0.14	0.05	1.00
	Far	0.46	0.39–0.55	0.35	0.00	0.31	0.68	1.00
13	Near	0.28	0.25–0.35	0.10	0.00	0.14	0.05	1.00
	Far	0.40	0.33–0.47	0.10	0.00	0.10	0.02	1.00
14	Near	0.48	0.39–0.53	0.12	0.00	0.10	0.00	1.00
	Far	0.66	0.54–0.74	0.14	0.05	0.09	0.39	1.00

CI, confidence interval; WR, Weber ratio. Deviance and R^2 are reported as goodness-of-fit measures.

Table S2. Results of analysis of variance in Experiment 1

	*df*1, *df*2	*F*	*p*	η_p^2
Group	1, 12	7.24	0.02	0.38
Task	2, 24	79.25	<0.001	0.87
Distance	1, 12	135.94	<0.001	0.92
Group * Task	2, 24	4.32	0.03	0.26
Group * Distance	1, 12	1.71	0.22	0.12
Task * Distance	1.19, 14.33	8.79	0.007	0.42
Group * Task * Distance	1.24, 28.54	0.82	0.40	0.06

Table S3. Psychometric function fitting metrics for the judgment task in Experiment 2.

Sub ID	Condition	PSE	95% CI	slope	lapse	WR	deviance	R^2
1	R	0.20	0.18–0.25	0.09	0.00	0.19	0.70	1.00
	SI	0.25	0.22–0.28	0.08	0.00	0.14	1.08	1.00
2	R	0.42	0.38–0.47	0.18	0.00	0.18	0.46	1.00
	SI	0.30	0.26–0.36	0.23	0.02	0.33	7.90	0.95
3	R	1.00	0.86–1.22	0.22	0.00	0.09	0.00	1.00
	SI	0.75	0.69–0.90	0.20	0.00	0.11	0.00	1.00
4	R	0.53	0.45–0.62	0.37	0.00	0.29	1.13	0.99
	SI	0.34	0.26–0.41	0.30	0.04	0.38	7.14	0.93
5	R	0.37	0.32–0.43	0.26	0.00	0.28	3.04	0.97
	SI	0.46	0.40–0.57	0.41	0.00	0.36	4.62	0.93
6	R	0.39	0.34–0.46	0.28	0.00	0.30	0.62	1.00
	SI	0.34	0.30–0.39	0.20	0.00	0.24	0.53	1.00
7	R	0.23	0.14–0.33	0.50	0.00	0.88	6.99	0.90
	SI	0.10	−0.15–0.13	0.18	0.00	0.72	5.27	0.85
8	R	0.34	0.30–0.40	0.14	0.03	0.18	2.35	1.00
	SI	0.32	0.28–0.37	0.18	0.00	0.23	0.49	1.00
9	R	0.31	0.25–0.36	0.26	0.00	0.35	1.97	0.98
	SI	0.28	0.24–0.34	0.22	0.00	0.31	2.49	0.98
10	R	0.72	0.52–0.99	1.07	0.00	0.61	7.12	0.82
	SI	0.51	0.39–0.73	0.75	0.08	0.67	8.92	0.87
11	R	0.46	0.42–0.52	0.15	0.00	0.14	0.01	1.00
	SI	0.38	0.34–0.42	0.10	0.00	0.11	0.05	1.00
12	R	0.18	0.15–0.21	0.09	0.00	0.20	0.82	0.99
	SI	0.20	0.17–0.24	0.09	0.00	0.20	2.52	0.99
13	R	0.27	0.24–0.32	0.16	0.00	0.25	0.34	1.00
	SI	0.43	0.38–0.51	0.27	0.00	0.26	2.64	0.98
14	R	0.39	0.32–0.47	0.38	0.00	0.42	2.18	0.99
	SI	0.25	0.21–0.31	0.19	0.00	0.33	0.45	1.00
15	R	0.65	0.54–0.69	0.13	0.00	0.08	0.00	1.00
	SI	0.46	0.40–0.54	0.30	0.00	0.27	2.14	0.98
16	R	0.41	0.34–0.47	0.25	0.00	0.25	4.69	0.96
	SI	0.35	0.31–0.41	0.27	0.00	0.32	2.05	0.99
17	R	0.23	0.20–0.28	0.14	0.02	0.26	3.83	0.99
	SI	0.34	0.26–0.43	0.30	0.04	0.39	7.14	0.93

18	R	0.25	0.18–0.33	0.39	0.00	0.63	4.54	0.95
	SI	0.24	0.20–0.30	0.21	0.00	0.36	0.56	1.00
19	R	0.28	0.20–0.34	0.34	0.00	0.50	8.48	0.90
	SI	0.25	0.22–0.32	0.11	0.04	0.20	6.01	0.98
20	R	0.24	0.21–0.29	0.14	0.00	0.24	0.44	1.00
	SI	0.29	0.24–0.38	0.20	0.05	0.30	5.16	0.98
21	R	0.26	0.23–0.31	0.14	0.00	0.22	1.62	1.00
	SI	0.26	0.22–0.32	0.29	0.00	0.46	4.36	0.97
22	R	0.44	0.39–0.53	0.20	0.04	0.20	1.89	0.99
	SI	0.34	0.26–0.41	0.38	0.00	0.46	4.07	0.97
23	R	0.56	0.48–0.64	0.32	0.00	0.23	1.97	0.99
	SI	0.48	0.44–0.55	0.10	0.00	0.08	0.05	1.00
24	R	0.26	0.23–0.31	0.15	0.00	0.23	0.18	1.00
	SI	0.25	0.21–0.30	0.18	0.00	0.30	1.59	0.99
25	R	0.27	0.22–0.32	0.24	0.00	0.36	3.02	0.98
	SI	0.27	0.23–0.30	0.09	0.00	0.15	0.25	1.00
26	R	0.42	0.37–0.49	0.24	0.00	0.23	1.81	0.99
	SI	0.40	0.35–0.46	0.20	0.03	0.21	4.03	0.98

CI, confidence interval; WR, Weber ratio. Deviance and R^2 are reported as goodness-of-fit measures.

Table S4. Results of analysis of variance in Experiment 2

	*df*1, *df*2	*F*	*p*	η_p^2
Group	1, 23	0.18	0.67	0.01
Task	1.24, 28.54	24.09	<0.001	0.51
Condition	1, 23	245.18	<0.001	0.91
Group * Task	1.24, 28.54	0.43	0.56	0.02
Group * Condition	1, 23	1.23	0.28	0.05
Task * Condition	1.23, 28.40	47.3	<0.001	0.67
Group * Task * Condition	1.23, 28.40	0.61	0.47	0.03

Table S5. Bayes factor (BF_{10}) for each target duration under each task condition in Experiment 3

	Reachability judgment	Color judgment	Control
0.1 s	71.2*	261.0*	55.9*
0.15 s	69.2*	22.8*	73.0*
0.2 s	38.1*	9.2*	24.0*
0.3 s	11.6*	9.5*	8.4*
0.5 s	0.8	0.5	1.2
0.7 s	0.5	0.3	0.3
1.0 s	6.6*	1.1	0.5

Asterisks indicate that the alternative hypothesis (that the reproduced duration was longer or shorter than the target duration) was supported ($BF_{10} > 3$).

Table S6. Psychometric function fitting metrics for the judgment task in Experiment 3.

Sub ID	PSE	95% CI	slope	lapse	WR	deviance	R^2
1	0.41	0.28–0.48	0.36	0.00	0.36	1.23	0.99
2	0.32	0.23–0.38	0.36	0.00	0.46	3.29	0.96
3	0.18	0.07–0.21	0.14	0.00	0.33	1.31	0.98
4	0.30	0.25–0.38	0.29	0.00	0.39	2.44	0.98
5	0.26	0.19–0.31	0.17	0.00	0.26	0.25	1.00
6	0.68	0.56–0.90	0.14	0.12	0.10	1.07	0.97
7	0.71	0.55–0.81	0.48	0.00	0.28	0.98	0.99
8	0.65	0.34–0.80	0.96	0.00	0.60	6.60	0.90
9	0.63	0.51–0.71	0.35	0.00	0.23	0.03	1.00
10	0.52	0.47–0.60	0.11	0.00	0.08	0.00	1.00
11	0.93	0.74–1.14	0.61	0.00	0.27	0.20	1.00
12	0.22	0.14–0.26	0.17	0.00	0.32	2.79	0.97

CI, confidence interval; WR, Weber ratio. Deviance and R^2 are reported as goodness-of-fit measures.

Table S7. Results of linear mixed-effects model analysis for the effect of temporal distance on the decision time in the judgment task

		Estimate	*df*	*t*	*p*
Exp1 (Near target)	temporal distance	-0.93	8.39	-8.45	< 0.001
	intercept	1.02	10.74	15.06	< 0.001
Exp1 (Far target)	temporal distance	-0.93	9.66	-8.53	< 0.001
	intercept	1.06	10.59	15.21	< 0.001
Exp2 (R condition)	temporal distance	-0.67	25.10	-7.90	< 0.001
	Intercept	0.97	23.82	20.87	< 0.001
Exp2 (SI condition)	temporal distance	-0.67	24.82	-6.89	< 0.001
	Intercept	1.07	25.06	18.87	< 0.001
Exp3	temporal distance	-0.49	95.64	-6.41	< 0.001
	Intercept	1.01	11.39	14.39	< 0.001

Linear mixed-effect model (LMEM) analyses were performed using the *lme4* package implemented in R (version 4.3.3; R Development Core Team, Vienna, Austria). The model was as follows: $lmer\ (decision\ time\ {\sim} Distance + (1 + Distance|\ participant))$, where *Distance* corresponds to the temporal distance. Both random intercepts and random slopes for *Distance* were specified for participants. Degrees of freedom were estimated using the Satterthwaite approximation implemented in the *lmerTest* package.

Table S8. Results of LMEM analysis for the effect of action TET on judgment TET using the data from Experiments 1 and 2, and control experiment.

	Estimate	*df*	*t*	*p*
action TET	0.59	85.92	3.87	< 0.001
Target distance	0.03	42.76	0.20	0.84
Condition	0.25	52.33	2.31	0.02
action TET: Target distance	-0.18	14.05	-1.58	0.14
action TET: Condition	-0.21	43.49	-0.86	0.40
Intercept	-0.01	86.0	-0.10	0.92

Degrees of freedom were estimated using the Satterthwaite approximation implemented in the *lmerTest* package.
